# Identification and functional characterization of *ORF19.5274*, a novel gene involved in both azoles susceptibility and hypha development in *Candida albicans*

**DOI:** 10.3389/fmicb.2022.990318

**Published:** 2022-10-03

**Authors:** Mingjiao Huang, Longbing Yang, Luoxiong Zhou, Chaoqin Sun, Wenjing Zhao, Jian Peng, Zhenlong Jiao, Chunren Tian, Guo Guo

**Affiliations:** ^1^The Key and Characteristic Laboratory of Modern Pathogen Biology, School of Basic Medical Sciences, Guizhou Medical University, Guiyang, China; ^2^Key Laboratory of Environmental Pollution Monitoring and Disease Control (Guizhou Medical University), Ministry of Education, Guiyang, China; ^3^Center of Laboratory Medicine, The Affiliated Hospital of Guizhou Medical University, Guiyang, China; ^4^Translational Medicine Research Center, Guizhou Medical University, Guiyang, China

**Keywords:** *Candida albicans*, *ORF19.5274*, azole resistance, ergosterol, *Galleria mellonella*

## Abstract

Azole resistance is becoming increasingly serious due to the frequent recurrence of fungal infections and the need for long-term clinical prevention. In our previous study, we discovered *ORF19.5274* with an unknown function by TMT^™^ quantitative proteomics technology after fluconazole (FLC) treatment of *Candida albicans*. In this study, we created the target gene deletion strain using CRISPR-Cas9 editing technology to see if *ORF19.5274* regulates azole sensitivity. The data showed that *ORF19.5274* was involved in hyphal development and susceptibility to antifungal azoles. Deleting this gene resulted in defective hyphal growth in solid medium, while only a weak lag in the initiation of hyphal development and restoring hyphal growth during the hyphal maintenance phase under liquid conditions. Moreover, intracellular reactive oxygen species (ROS) assay and propidium iodide staining assays showed increased endogenous ROS levels and membrane permeability, but decreased metabolic activity of biofilm in *orf19.5274Δ/Δ* after treatment with FLC in comparison with either SC5314 or *orf19.5274Δ/Δ::ORF19.5274* strains. More importantly, *orf19.5274Δ/Δ* significantly enhanced the FLC efficacy against *C. albicans* in infected *Galleria mellonella* larvae. The above characteristics were fully or partially restored in the complemented strain indicating that the changes caused by *ORF19.5274* deletion were specific. In summary, the *ORF19.5274* gene is required for hyphal development of *C. albicans*, and is correlated with the response to antifungal azoles *in vitro* and *in vivo*. The identification of *ORF19.5275* is promising to expand the potential candidate targets for azoles.

## Introduction

*Candida albicans* is an important opportunistic pathogenic fungus that can cause diverse diseases, ranging from superficial mucocutaneous candidiasis to life-threatening systemic disseminated infections in immunodeficient individuals (Lok et al., 2021). Its frequent infection rate and high mortality rate are dramatically challenging for the clinical treatment. Triazole drugs are the most widely used in clinics because of their superior oral bioavailability, wide antifungal spectrum, and low toxic side effects (Lu et al., 2021). These drugs exert their effects on fungal growth mainly by repressing the lanosterol-14α-demethylase (*Erg11p*) and blocking the biosynthetic pathway of ergosterol in the fungal cell membrane, which causes changes in cell membrane permeability and induce leakages of important intracellular components (Brunetti et al., 2019). Perioperative pharmacotherapy and long cycles of pharmacotherapy in patients with candidiasis have led to the development of clinical resistance to azoles as a major cause of treatment failure (Berman and Krysan, 2020). Therefore, it is very urgent to elucidate the mechanism of resistance of *C. albicans* and solve the problem of clinical azole resistance. Current studies have found that the resistance mechanisms of *C. albicans* to azoles mainly involve the following aspects: first, efflux pump gene overexpression; second, variation or high expression of azole target enzyme genes; third, biofilm formation; fourth, reduced azole permeability of strains, and so on (Nishimoto et al., 2020; Lee et al., 2021; Fisher et al., 2022). Genes related to drug resistance mainly include the ATP-binding cassette (ABC) transporter superfamily encoded by *CDR1* or *CDR2*, the major facilitator (MF) superfamily encoded by *MDR1*, the azole target genes (ERGs) in the ergosterol synthesis pathways, and the pleiotropic drug resistance genes (PDR), etc. (Akins, 2005; Singh et al., 2018; Whaley et al., 2018; Moreno et al., 2019). Drug resistance is a complex process involving multiple factors and levels, which has not yet been completely clarified. In particular, new drug resistance genes and mechanisms are still unclear.

Many differentially expressed proteins with uncharacterized functions were identified by TMT^™^ quantitative proteomics in *C. albicans* after FLC treatment. Among them, the protein encoded by *ORF19.5274* was significantly upregulated and it seemed this gene was related to FLC sensitivity. The bioinformatics analysis revealed that the length of *ORF19.5274* is 3,066 bp, encodes 1,021 amino acids, and contains a pleckstrin homology (PH) or phosphotyrosine binding (PTB) domain (Fidler et al., 2016), which is homologous to the *SKG3* (Suppressor of the lethality of KEX2-GAS1 double null mutant protein 3) protein in *S. cerevisiae*. Although *S. cerevisiae* is a model organism, the functional characterization of this protein is limiting. Furthermore, the alias of the *ORF19.5274* gene in *C. albicans* is *CAF120* and KEGG analysis suggests it may be involved in mRNA degradation processes as a transcription factor (TF; Tomishige et al., 2005). *CAF120*, one of the subunits of the CCR4-NOT transcription complex, is a universal TF in the nucleus and may be a primary mRNA deadenylase involved in mRNA conversion in the cytoplasm. Furthermore, the NOT protein subcomplex regulates the transcription of many genes, which can directly or indirectly inhibit general transcriptional mechanisms (Chen et al., 2001; Mulder et al., 2005, 2007; Kruk et al., 2011; Collart et al., 2013; Castelli et al., 2015). Therefore, *ORF19.5274* may also function as a TF in the vital activities of *C. albicans*. TFs often play crucial roles in the mechanisms of antifungal resistance. For instance, mutations in TFs genes such as *Ndt80p*, *Efg1p*, *Upc2p*, *Mrr1p* can alter sensitivity to azoles by modulating the expression of efflux pump genes or azole target enzyme genes in *C. albicans* (Morschhäuser et al., 2007; Heilmann et al., 2010; Lo et al., 2015; Feng et al., 2019). Identification of novel genes associated with antifungal responses is conducive to tapping into novel antifungal targets. In this study, an *orf19.5274Δ/Δ* was established through the CRISPR-Cas9 gene editing technique, and the partial functions of this new gene in the life activities of *C. albicans* were reported. Unlike the function of the homologous gene in *S. cerevisiae* (Drees et al., 2001; Suzuki et al., 2003; Ubersax et al., 2003), we found no significant changes in sensitivity to cell wall stress and no reduction or deposition of chitin in the mutant. Interestingly, hyphal development of the deletion strain behaved inconsistently under solid and liquid hyphae-inducing conditions and the mutant showed increased sensitivity to azoles, as well as increased intracellular ROS levels and FLC-dependent membrane permeability. Furthermore, the data showed that deletion of *ORF19.5274* could significantly improve the efficacy of FLC in a larval infection model, indicating that the gene is likely to be a new effector correlated with antifungal responses, which provides a reference and experimental basis for the development of new therapeutic strategies and new targets for azole drugs.

## Materials and methods

### Strains and plasmids

The *Candida albicans* SC5314 used in this study was donated by Dr. Zhenbo Xu (South China University of Technology). Hernday Laboratory provided pADH99 and pADH100 (Nguyen et al., 2017); Takara Corporation provided DH5; and other fungal strains were created in our laboratory. These strains were revitalized in YPD broth (Solarbio, Beijing, China) at 30°C for 16–18 h with 200 rpm/min shaking. The genotypes of each strain are shown in Table 1.

**Table 1:** *Candida albicans* strains used in this study.

Strains	Genotype	References
SC5314	Wild type	
*orf19.5274Δ/Δ*	*orf19.5274Δ/orf19.5274Δ*	This study
*orf19.5274Δ/Δ::ORF19.5274*	*orf19.5274Δ/orf19.5274Δ::ORF19.5274*	This study

### Construction, identification and complementation of deficient strain

**Figure 1:**
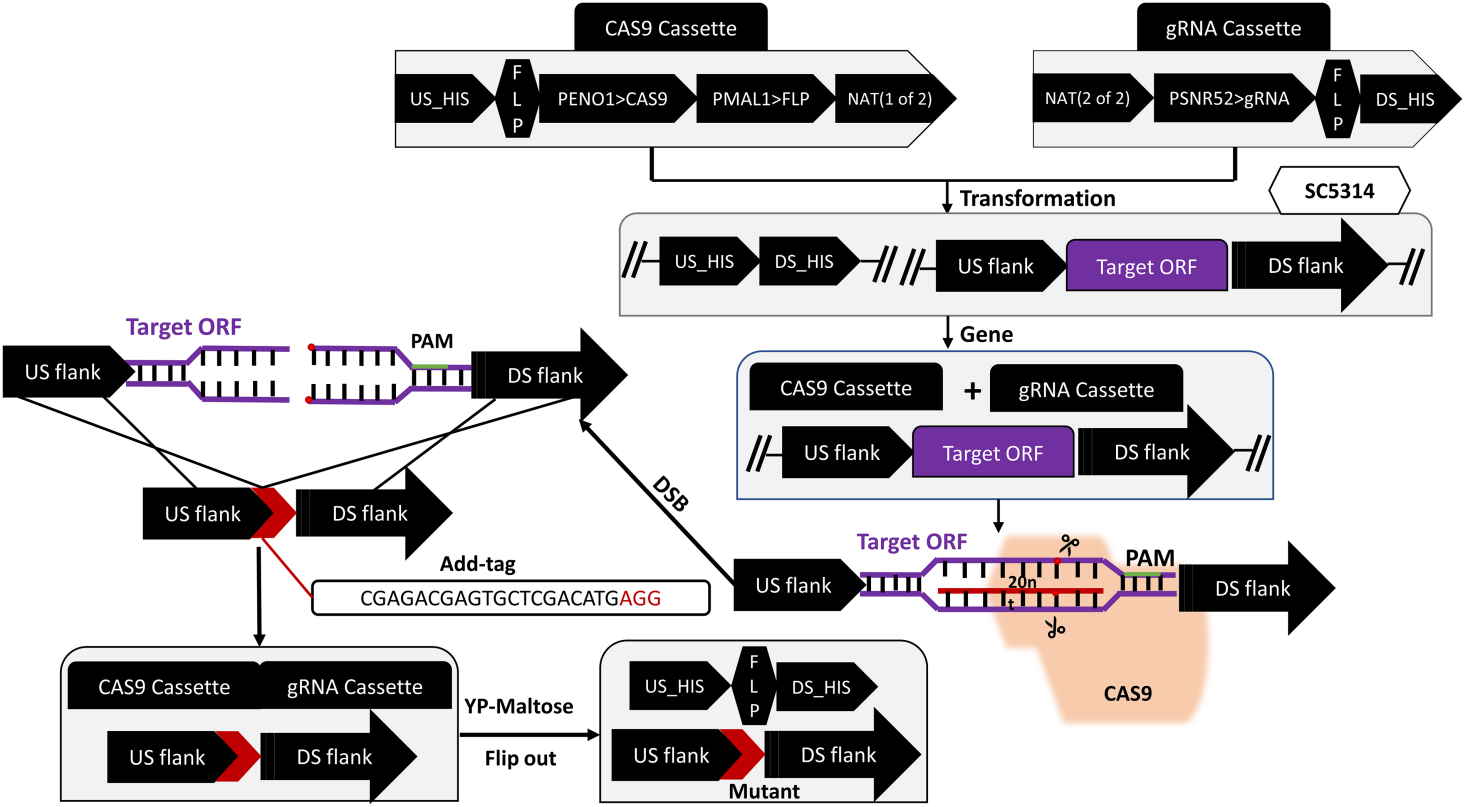
Schematic diagram of target gene knockout strategy.

The target gene was knocked out according to the *CRISPR/Cas9* gene editing technique (the flowchart is shown in Figure 1) developed by Nguyen et al. (2017). A 20 bp gRNA target sequence was designed using the online software Benchling^1^ with the “Design and Analyze Guides” tool. Subsequently, the following flanking sequence was added to both ends of the designed gRNA: 5′-CGTAAACTATTTTTAATTTG (gRNA) GTTTTAGAGCTAGAAATAGC-3′, which was sent to Shanghai Sangon for synthesis. The pADH100-gRNA expression cassette was constructed by inserting the designed 20 bp gRNA into the successfully linearized pADH100 expression plasmid using Circular Polymerase Extension Cloning (CPEC). Using the Overlap-PCR method, a DNA sequence of about 500 bp upstream and downstream of the target gene was selected as the repair template, and forward and reverse primers were designed on both sides of the sequence to amplify a donor DNA band of about 1,000 bp by ligation with the ADD-TAG sequence. The plasmids pADH99 and pADH100-gRNA were digested with the FastDigest MssI enzyme and then transfected into SC5314 competent cells with homologous flanks. For the complemented strain, SC5314 was amplified using the flanking specific primers of the target gene. A revertant plasmid containing a 20 bp ADD-TAG sequence was inserted into competent cells of the knockout strain and co-transfected with the pADH99 plasmid. Single colonies with successfully deleted and restored target genes were induced with YPM medium to produce FLIP recombinase, thereby removing the labels of CRISPR components and NAT marker cassettes.

Single colonies were inoculated into 5 ml of YPD broth, shaken for 12 h (30°C, 200 rpm/min), centrifuged, and washed three times with sterile PBS. The concentration of the suspension was adjusted to 2.0 × 10^6^ CFU/ml using a modified cow abalone count plate, and the suspension with or without FLC was co-cultured in an incubator at 30°C. At 2 h intervals, the absorbance values were measured at OD_630_ nm with an iMark^™^ microplate reader, and then the time-growth curve was plotted.

### Hyphal induction testing

*Candida albicans* cultures at the logarithmic growth stage were adjusted to 2.0 × 10^6^ CFU/ml with YPD + 10% FBS and Spider liquid inducing medium, respectively. The adjusted suspension was added to a 24-well plate and incubated at 37°C for 2 and 5 h, respectively.

*Candida albicans* cultures at the logarithmic growth stage were adjusted to 2.0 × 10^6^ CFU/ml with sterile PBS buffer. 10 μl cell suspension was dropped on YPD + 10% FBS, Spider, or Lee’s solid medium and cultured at 37°C for 5 days. The growth of hyphae on inducing mediums was observed under an inverted microscope and photographed.

### Drug susceptibility testing

*Candida albicans* cultures at the logarithmic growth phase were taken and resuspended with YPD broth and adjusted to 2.0 × 10^3^ CFU/ml. Drug susceptibility tests were performed according to the American Clinical and Laboratory Standards Institute (CISL) recommendations M38-A2 (Mahlapuu et al., 2020) and M27-A3 (Walkenhorst et al., 2013). 96-well plates were inoculated with 100 μl drug as well as 100 μl cell suspension and cultured at 30°C for 48 h. The OD_630_ values of each well were measured by the iMark^™^ microplate reader and supplemented by visual inspection. The minimum inhibitory concentration (MIC) value of the tested drug was determined as the minimum inhibitory concentration in the wells where no growth was observed visually compared to the negative control. This assay was repeated three times with three technical replicates each time.

*Candida albicans* cultures at the logarithmic growth phase were collected, and the fungal cell concentration was adjusted to 1.0 × 10^7^ CFU/ml with YPD liquid medium, and then the cell suspension was diluted to five concentration gradients with sterile PBS in multiple ratios. Three μl of fungal suspension was dripped onto each drug-containing YPD solid plate. After the plates were dried, inverted and cultured in a 30°C incubator for 48 h. The growth status of the colonies was observed and photographed.

### Biofilm assay

Absorbance values and Confocal Laser Scanning Microscope (CLSM) images were used to quantify and visualize the biofilms in *C. albicans*, respectively. The effect of mutants on biofilms was measured through a 2,3-bis(2-methoxy-4-nitro-5-sulfophenyl) 2H-tetrazolium-5-carboxanilide sodium salt (XTT) reduction assay. In brief, 100 μl *C. albicans* cell solution at the density of 2 × 10^6^ CFU/ml was incubated in RPMI 1640 medium in a 96-well flat-bottomed polypropylene plate at 37°C for 90 min for initial adhesion. Nonadherent cells were removed and fresh RPMI-1640 medium with or without different concentrations of FLC was added, followed by incubation for 24 h. For examination of FLC on mature biofilms, biofilms pre-grown for 24 h were exposed to different concentrations of FLC and incubated for another 24 h at 37°C. After incubation, the unadhered cells were washed off with sterile PBS. 100 μl newly prepared XTT/menadione (10 mM; Shanghai yuanye, China) solution was added and incubated for 3 h at 37°C in the dark, then detected at OD_490_ using an iMarkTM microplate reader. The assay was performed in triplicate. Finally, the percent of biofilm metabolic activity normalized to the no-treatment control was calculated as follows:


$$\begin{array}{l} BiofilmMetabolicActivity\;\left(\%\right)\\ =\left(\frac{A_{490}^{treated}-A_{490}^{medium}}{M\left(A_{490}^{untreated}-A_{490}^{medium}\right)}\right)\times 100\% \end{array}$$

Where $A_{490}^{\mathrm{treated}}$ is the mean absorbance for wells containing FLC, $A_{490}^{\mathrm{untreated}}$ is the mean absorbance of untreated cells, and $A_{490}^{\mathrm{medium}}$ is the mean absorbance of wells containing medium. One-way ANOVA was used for data statistics.

Biofilm samples were photographed by CLSM and prepared as described below. Briefly, sterile polylysine cell-attached slides were placed in 24-well flat-bottom polypropylene plates and incubated with 500 μl *C. albicans* cell solution at a density of 2 × 10^6^ CFU/ml in RPMI 1640 medium at 37°C for 90 min for initial adhesion. Nonadherent cells were removed and fresh RPMI-1640 medium with or without different concentrations of FLC was added, then incubated for 24 h. Pre-grown biofilms were exposed to different concentrations of FLC and incubated for another 24 h at 37°C to check for FLC on mature biofilms. The well plates were then treated with propidium iodide (PI; 10 μg/ml) and SYTO9 (10 mM) and co-incubated for 15 min at 37°C in the dark before being washed with PBS. Next, clip out the cell-attached slides inside the well plates with sterile tweezers and pour them onto the sterile glass slide immediately. Finally, images were taken using the Olympus CLSM with an excitation wavelength of 488 nm for SYTO9 and 525 nm for PI excitation.

### Investigation of cell membrane permeability by PI staining

The concentration of *C. albicans* cells was adjusted to 2.0 × 10^6^ CFU/ml with YPD broth containing different concentrations of FLC. A total of 1.5 ml YPD broth with cells was transferred to diverse tubes and shaken at 200 rpm for 8 h. Cells were centrifuged to remove the medium and washed three times with sterile PBS. Untreated and treated cells were stained for 30 min at 30°C with 50 μg/ml PI (Sigma, United States). After that, cells were washed three times with sterile PBS, and the percentage of PI-positive cells was rapidly detected by flow cytometry (Beckman Coulter). Excitation wavelength/emission wavelength: 535/615 nm.

### Assays on the level of intracellular reactive oxygen species (ROS)

In order to analyze whether the redox reaction of the mutant was changed and to observe whether the oxidative stress response of the mutant to FLC was different, ROS levels were examined using 2′, 7′-dichlorofluorescein diacetate (DCFH-DA). Briefly, 2.0 × 10^6^ CFU/ml of *C. albicans* at exponential phase with YPD broth containing different concentrations of FLC was collected. After incubation for 8 h, the fungal cells were washed using sterile PBS, adjusted to 2.0 × 10^6^ CFU/ml and then stained with 10 μM DCFH-DA for 30 min at 30°C in the dark. Next, the samples were washed twice using PBS, followed by suspending the cells. Finally, the fluorescence intensity was detected by flow cytometry (Beckman Coulter; excitation wavelength/emission wavelength: 485/530 nm).

### RNA isolation and quantitative real-time PCR

Total RNA was extracted by the following method: *C. albicans* yeast cells and hyphal cells were collected by centrifugation, and 100 μl Trizol reagent was added followed by violent oscillation and mixing. The mixture was subjected to repeated freeze–thawing using liquid nitrogen until the cell wall was broken. Subsequently, 700 μl Trizol was added followed by incubation on ice for 20 min. Two hundred μl trichloromethane was added with vortexing for 15 s, followed by incubation on ice for 15 min, centrifuging at 13,000 rpm/min for 10 min, and aspirating the upper aqueous phase into a new 1.5 ml EPP tube. An equal volume of isopropanol was added to the tube, followed by incubation on an ice bath for 15 ~ 20 min. RNA was precipitated by centrifuging at 13000 rpm/min for 10 min. 75% ethanol was added to wash the precipitate, and an appropriate amount of DEPC water was added to dissolve RNA. Then 5 μl RNA solution was taken for electrophoresis after determining the concentration with a NanoDrop 2000 ultra-violet spectrophotometer. When the 28S and 18S ribosomal subunit bands of RNA samples were clear, the extracted RNA was found to be intact and could be used for subsequent reverse transcription. RNA reverse transcription cDNA was obtained according to the Thermo Fisher (Shanghai Yisun Biotechnology Co., Ltd.) reverse transcription kit. qRT-PCR was performed in triplicate and repeated in three independent experiments using an iQ5 Real-time PCR system (Bio-Rad). Primers used in qRT-PCR are listed in Supplementary Table S1. Independent reaction mixtures were carried out by the same cDNA for both the genes of interest and the β-actin using the SYBR Green agent (Thermo Fisher) according to the instructions. Reaction conditions: 50°C, 2 min; 98°C, 30 s; 98°C, 15 s; 60°C, 15 s; 72°C, 1 min; From Step 3 to 5, 1 min; 98°C, 30 s; 98°C, 15 s; 60°C, 15 s; 72°C, 1 min; From Step 3 to 5 for 40 cycles; Dissolution curves were performed using the instrument’s own program.

### Virulence and efficacy studies of *Galleria mellonella* larvae

Virulence test: The *G. mellonella* larvae were selected for survival and efficacy tests to investigate whether there were differences in virulence and FLC efficacy between the wild strain and the knockout strain. Here, healthy larvae without black spots, weighing between 250 and 300 mg, were randomly selected and divided into four groups (control group, SC5314 group, *orf19.5274Δ/Δ* group, *orf19.5274Δ/Δ::ORF19.5274* group), ten larvae in each group, and the concentration of the fungal solution was adjusted to 1.0 × 10^8^ CFU/ml with sterile PBS. The larvae were injected with 10 μl the fungal solution from the left lower limb of the larvae using a microscopic sterile syringe, and the survival or death of each group of larvae was subsequently observed.

Efficacy test: The samples were grouped as in the virulence test, and 1 h after injection of the fungal solution, 10 μl 64 μg/ml FLC solution was injected from the right lower limb of the larvae using a microscopic sterile syringe, and the sterile PBS plus drug group was used as a negative control. The larvae of each group were placed in clean Petri dishes and incubated in a 37°C incubator at 12-h day-night intervals. The survival or death of each larva was recorded every 24 h for 10 days, and any larvae that did not move or respond to light exposure were recorded as dead. Experiments were performed in three independent replicates, and the data was tallied as the sum of the data from the three experiments. Mortality rates were compared using the GraphPad 8.0 Log-Rank test.

### Statistical analysis

Every experiment was independently performed at least three times, and the data was expressed as mean ± SD of three independent experiments. Differences between experimental groups were assessed for significance using One-Way ANOVA with GraphPad Prism 8 software. For the *G. mellonlla* survival experiment, Long-rank was used in the analysis of Mantel-Cox survival curves. The ^*^p < 0.05，^**^p < 0.01 and ^***^p < 0.001 levels were considered to indicate statistical significance.

## Results

### Successful construction of *orf19.5274Δ/Δ* and complementary strains

**Figure 2:**
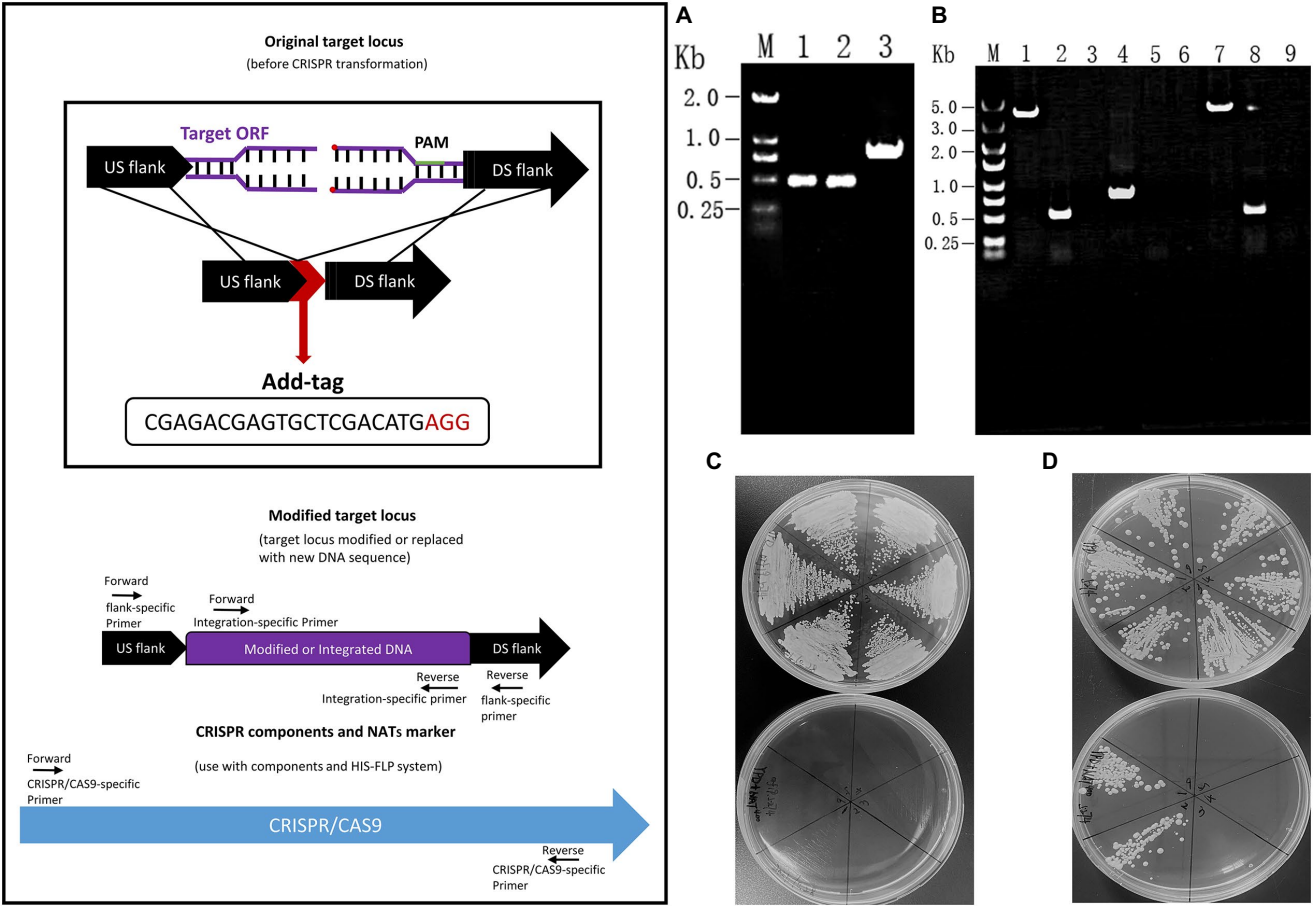
Construction and identification of *orf19.5274Δ/Δ* and *orf19.5274Δ/Δ::ORF19.5274* S trains. **(A)** Designing and identifying repair homology flanks. M: DL2000 Marker; 1–2: *orf19.5274*_Up_F/R and Down_F/R amplification products; 3: *orf19.5274* _Up_F/Down_R amplification products; **(B)** Colony PCR verification of SC5314, *orf19.5274Δ/Δ, orf19.5274Δ/Δ::ORF19.5274*. M: DL5000 Marker; 1–3: PCR products of SC5314 amplified by *ORF19.5274* gene flanker, ORF, and CRISPR-specific primers, respectively; 4–6: PCR products of *orf19.5274Δ/Δ* amplified by flanker, ORF, and CRISPR-specific primers, respectively; 7–9: PCR products of *orf19.5274Δ/Δ::ORF19.5274* by flanking, ORF, and CRISPR specific primers, respectively; **(C)** Flip-out removal of CRISPR components and NATs marker in *orf19.5274Δ/Δ* strain. Single colonies of *orf19.5274Δ/Δ* were grow on YPD solid plates containing with or without NAT^400^ for 2 days, the colonies that successfully removed the CRISPR component and NAT markers were able to grow on the YPD solid plates, but not on the YPD solid plates containing NAT^400^; **(D)** Flip-out removal of CRISPR components and NATs marker in *orf19.5274Δ/Δ::ORF19.5274* strain. Single colonies of *orf19.5274Δ/Δ::ORF19.5274* were grow on YPD solid plates containing with or without NAT^400^ for 2 days.

DNA fragments of about 500 bp each from the upstream and downstream of the *ORF19.5274* gene were linked by the Overlap-PCR method. The identification of the upstream and downstream homologous flanks of the gene and the repair homologous flank are shown in lanes 1, 2, and 3, respectively. There are bright bands at 500 and 1,000 bp (Figure 2A), which are consistent with the expected results, indicating the successful repair of homology flanks. The plasmids pADH99 and pADH100-gRNA were digested with the FastDigest MssI enzyme, then transformed into the competent cells of SC5314 and *orf19.5274Δ/Δ*, together with homologous flanks of knockout and complement strains. Colony PCR of the parental and complemented strains amplified 3,957 and 531 bp products by *ORF19.5274* gene flanking specific primers and ORF specific primers, respectively, while the knockout strain amplified 891 bp products and no ORF target band, respectively, indicating that *ORF19.5274* was absolutely deleted. Meanwhile, no bands were amplified by CRISPR-specific primers, meaning that the labels and NAT resistance cassettes were successfully removed from all three strains (Figure 2B). The fact that the NAT resistance cassettes were removed was also verified by streaking the colonies in YPD agar containing NAT (Figures 2C,D).

### Mutant strain increased sensitivity to FLC by downregulating ergosterol synthesis-related genes

To examine the changes in susceptibility to antifungal drugs resulting from *ORF19.5274* deletion, the MIC values of each strain were determined by the micro liquid dilution method. The MIC values of the mutant decreased 2-fold for FLC and 4-fold for KCZ, while the MIC values of the remaining antifungal drugs did not change in comparison with the parental and complemented strains (Table 2), indicating that the *ORF19.5274* deletion could affect the susceptibility to azole drugs.

**Table 2:** Antifungal susceptibility test of antifungal agents against *Candida albicans* strains.

Strains	MIC (μg/ml)				
	FLC	KCZ	CAS	AmB	5-FC
SC5314	4	0.25	0.25	0.5	2
*orf19.5274Δ/Δ*	2	0.0625	0.25	0.5	2
*orf19.5274Δ/Δ::ORF19.5274*	4	0.25	0.5	0.5	2

MIC, Minimal inhibitory concentration; FLC, Fluconazole; KCZ, Ketoconazole; CAS, Caspofungin; AMB, Amphotericin B; 5-FC, 5-Fluorocytosine.

**Figure 3:**
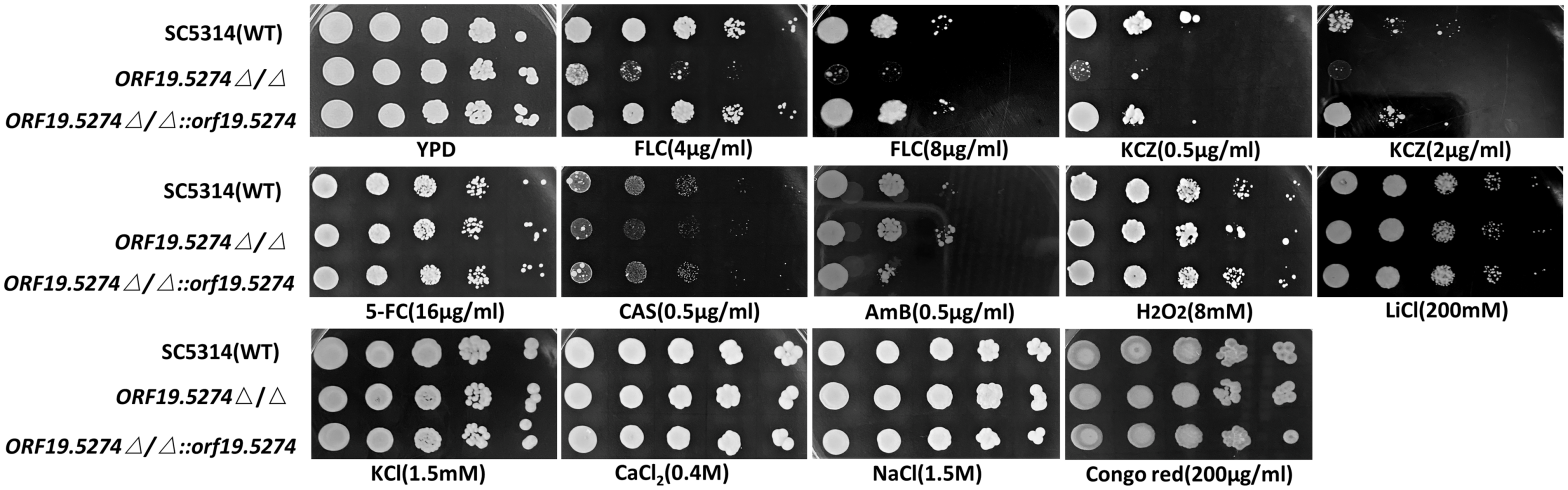
Spot assay to determine the sensitivity of *Candida albicans* to antifungal agents.

**Figure 4:**
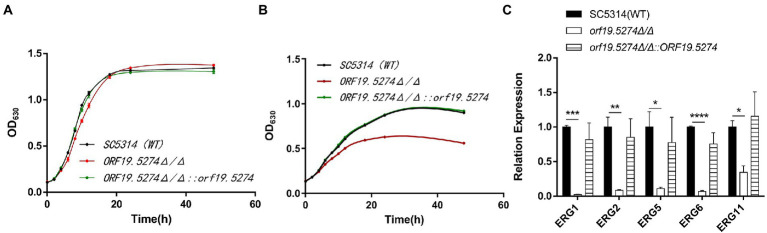
Mutant strains increase sensitivity to FLC by down-regulating ergosterol synthesis-related genes. **(A)** Growth and reproduction of wild, knockout, and complement strains within 48 h; **(B)** Growth and reproduction of wild, knockout, and complement strains within 48 h after FLC treatment with 2MIC concentration; and **(C)** Expression of ergosterol-related genes in SC5314, mutant, and revertant strains, respectively. The expression of the above genes in SC5314 was normalized, the remaining strains were controlled by the wild-type, and the 2^-ΔΔCt^ values were calculated. Three replicates were performed for each gene.

Solid spot assays were used to verify the susceptibility of mutants to antifungal drugs and other ions. The growth of the mutant strain was significantly inhibited on antifungal plates containing FLC and KCZ compared with the parental and complemented strains (Figure 3). This suggested that the susceptibility of the deletion strain to the cell membrane inhibitors FLC and KCZ was increased, which was consistent with the results of the microtitration dilution method. The susceptibility of the mutant to FLC in liquid medium was only reduced by 2 × MIC, probably because SC5314 itself is a FLC-sensitive strain with high sensitivity to FLC, and the growth of the fungal solution at low concentrations (~ 10^3^ CFU/ml) was more restricted. To confirm that the mutant strain did have increased susceptibility to FLC in the liquid drug assay, we dynamically monitored the growth and reproduction of the mutant after treatment with FLC (~ 10^6^ CFU/ml). There was no significant difference in the growth rate between the mutant and wild strains (Figure 4A), while the cell growth of the mutant was significantly inhibited after the addition of FLC (Figure 4B).

As previously described, deletion of the *ORF19.5274* increased susceptibility to antifungal azoles, prompting us to investigate the expression of genes involved in susceptibility to antifungal drugs. To this end, we used qRT-PCR to detect two major efflux pump genes and FLC target enzyme-related genes. It was found that deletion of this gene did not affect the expression of efflux pump genes that are associated with drug resistance (Supplementary Figure S3). Notably, the expression of ergosterol synthesis pathway-related genes (*ERG1*/*ERG2*/*ERG5*/*ERG6*/*ERG11*) was all decreased (Figure 4C), and it was hypothesized that *ORF19.5274* deletion mainly affected the sensitivity to azoles by downregulating ERG-like genes.

### *ORF19.5274* may be involved in liquid and solid hyphal development through different mechanisms

**Figure 5:**
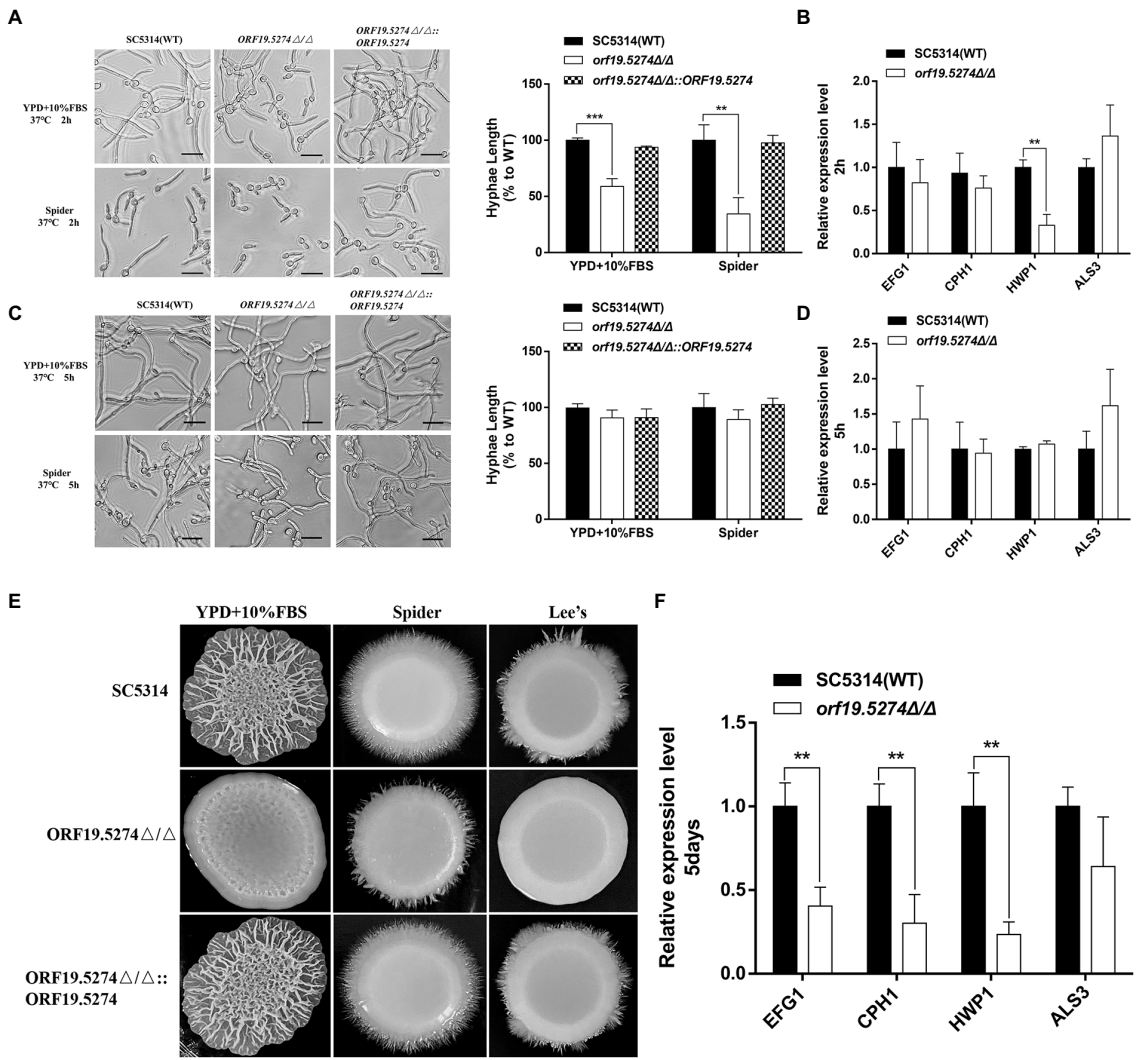
Morphological switching ability of *C. albicans* under filament-inducing conditions. **(A)** Cells were incubated in liquid YPD + 10%FBS serum and Spider medium for 2 h at 37°C. The samples were examined and photographed by light microscopy. Hyphae lengths in the wild-type, *orf19.5274Δ/Δ*, *orf19.5274Δ/Δ::ORF19.5274* strains were measured by a measurement tool in OlyVIA version 3.3. **(B)** After 2 h of incubation in the liquid inducing medium of Spider, cells were harvested and used for RNA isolation. Quantitative expression levels were determined by real-time PCR assays. **(C)** Cells were incubated in liquid YPD + 10%FBS serum and Spider medium at 37°C for 5 h, respectively. The samples were photographed by inverted microscopy. Hyphae lengths in the SC5314, *orf19.5274Δ/Δ*, *orf19.5274Δ/Δ::ORF19.5274* strains were measured by a measurement tool in OlyVIA version 3.3. **(D)** After 5 h of incubation in the liquid inducing medium of Spider, cells were harvested and used for RNA isolation. Quantitative expression levels were determined by real-time PCR assays. **(E)** SC5314, *orf19.5274Δ/Δ*, *orf19.5274Δ/Δ::ORF19.5274* strains were resuspended to an OD600 of 0.1 and spotted onto solid cells in YPD + 10%FBS serum, Spider, and Lee’s medium for 5 days at 37°C. **(F)** After 5 days of growth in solid Spider medium, cells were harvested mechanically in cold PBS buffer and used for RNA extraction. The expression analyses of selected hypha-specific genes were assessed by quantitative real-time PCR, and the data is representative of three independent experiments.

To study the role of *ORF19.5274* deletion in *C. albicans* morphogenesis, hyphal growth of the mutant in different hyphal induction media was observed. Our data showed that at the initial stage of hyphal development (2 h), the hyphal length of the mutant was shorter than the wild and complementary strains (Figure 5A, right). However, in the hyphal maintenance phase (5 h), the length difference of the hyphae recovered (Figure 5C, right). These results supported the hypothesis that the *ORF19.5274* deletion had a slight lag in the initiation phase of the hyphae under liquid conditions without affecting hyphal growth in the maintenance phase. Genes directly or indirectly related to hyphae formation include *HWP1*, *ALS3*, *CPH1* and *EFG1* (Staniszewska et al., 2015; Haque et al., 2016; Glazier, 2022), which are part of the core filamentation reaction. As long as *C. albicans* forms hyphae, their expression will be induced (Martin et al., 2013; Ruben et al., 2020). It has been reported that a decrease in hyphal-related gene expression leads to inhibition of *C. albicans* hyphae formation (Haque et al., 2016). In order to further explore the possible mechanism of the changes in the transcription level of hyphal-specific genes under liquid induction conditions, real-time PCR technology was used to determine the transcription level of hyphal-specific genes in liquid-induced media. Consistent with the phenotype, the deletion of *ORF19.5274* inhibited the expression of some hyphal-specific genes at the initial phase of hyphae development (Figure 5B), but did not affect the expression of hyphae-specific genes in the hyphal maintenance phase (Figure 5D), indicating that the effect of *ORF19.5274* on liquid hyphae growth of *C. albicans* only played a role in the early stages.

Subsequently, we analyzed the phenotype of the mutant under different solid hyphal induction conditions at 37°C. The *ORF19.5274* deleted strain was found to be defective in hyphae formation on YPD + 10% FBS, Spider, and Lee’s solid inducing mediums compared with the parental and complementary strains. The wild-type and revertant strains formed larger folded colonies with infiltrative hyphae growing at the edges, while the mutant formed smooth and flat colonies with almost no hyphae at the edges (Figure 5E). To understand the reasons for the hyphal defects in solid medium, total RNA was extracted and the mRNA level of hyphal-specific genes was analyzed. Our data suggested that *ORF19.5274* deletion significantly reduced the expression of hyphal-specific genes under solid inducing conditions (Figure 5F). The *ORF19.5274* deletion strain may have impaired transcriptional activity under liquid and solid-inducing conditions.

### *Orf19.5274Δ/Δ* strengthened the ability of FLC to inhibit biofilm formation in *Candida albicans*

**Figure 6:**
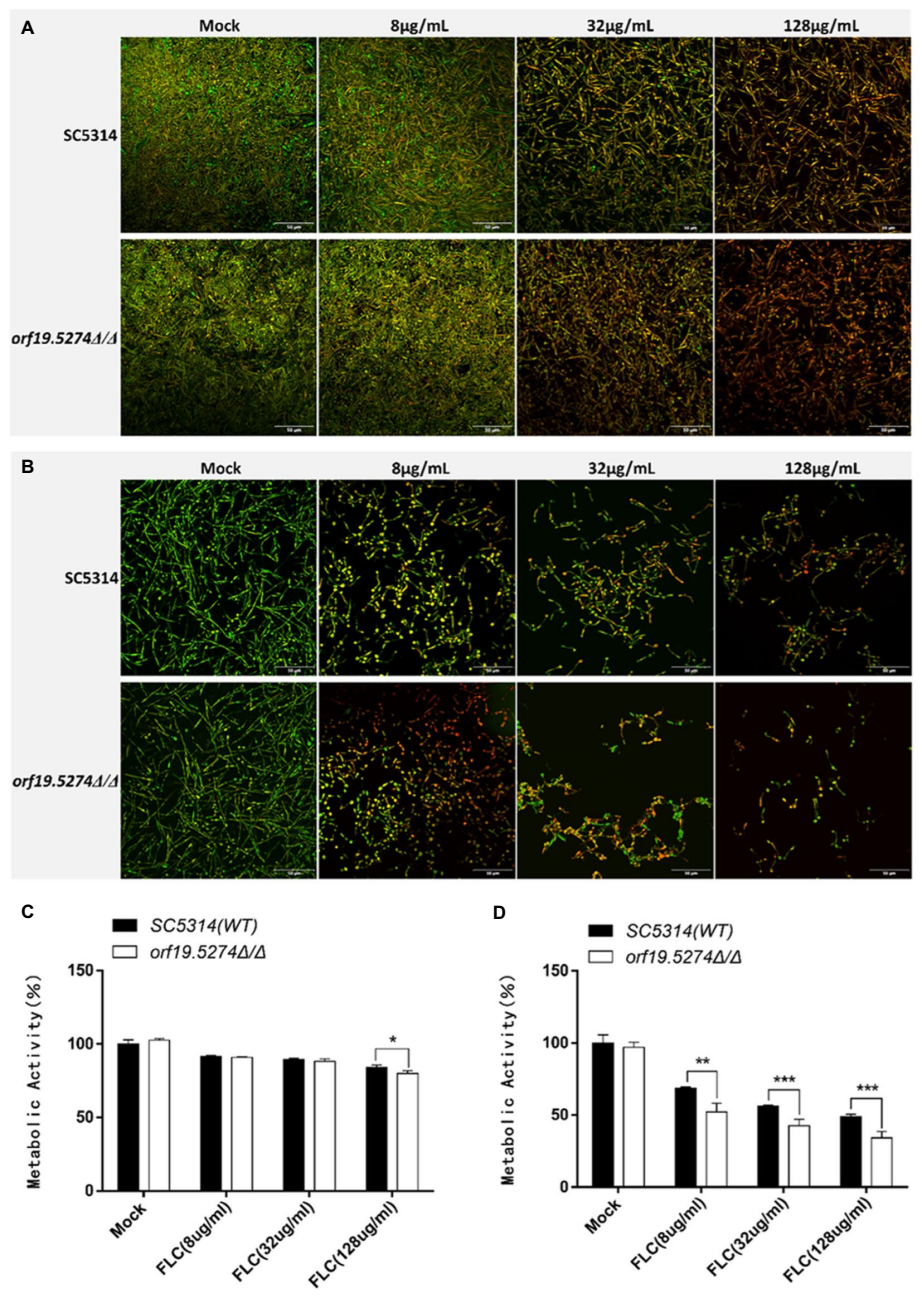
Effect of *orf19.5274Δ/Δ* on *Candida albicans* biofilm. **(A)** CLSM images of mature biofilm treated with various FLC concentrations for 24 h. The biofilms were stained with viability indicators (SYTO9 and PI). Live cells stain green, and dead cells are red or yellow-colored. **(B)** CLSM images of biofilm formation after 24 h of co-culture with different concentrations of FLC and *C. albicans*; **(C)** XTT assay to compare FLC’s metabolic activity in mature biofilm between mutant and wild strains. *C. albicans* cells were incubated in RPMI-1640 medium at 37°C for 24 h to form a mature biofilm. Next, the supernatant was discarded. **(D)** XTT assay to compare the metabolic activity of FLC in the biofilm formation process between mutant and wild strains. For biofilm formation, *C. albicans* cells were incubated with FLC (8 μg/ml, 32 μg/ml and 128 μg/ml) in RPMI-1640 medium at 37°C for 24 h. XTT reduction was performed to determine the level of biofilm formation at various treatments, and colorimetric absorbance was measured at 490 nm. Error bars represent the standard deviation of four independent experiments. In comparison to the parent strain SC5314, *p < 0.05, **p < 0.01, ***p < 0.001.

The impact of *ORF19.5274* deletion on biofilm formation, FLC to eliminate mature biofilm, and inhibition of biofilm formation were investigated by confocal laser scanning microscopy and XTT reduction assays, respectively. The metabolic activity of the biofilm in the mutant was basically the same as that of the parental strain SC5314 (Figure 6-mock group, *p* > 0.05), indicating that deletion of the *ORF19.5274* gene did not affect the biofilm formation of *C. albicans*. After 2 h of FLC action on the mature biofilm, the ability of FLC to sweep away the mature biofilm was poor at all concentrations, but the ability to clean the mature biofilm in the mutant was enhanced (*p < 0.05*) at the highest concentration (128 μg/ml) compared with that of the parental strain (Figure 6C). Meanwhile, the PI-positive cells were increased at the highest concentration of FLC shown in the images (Figure 6A). Additionally, FLC significantly inhibited biofilm formation in *C. albicans* after co-cultivation with 8, 32, and 128 μg/ml FLC, respectively, for 24 h. More importantly, the ability to inhibit biofilm formation in the mutant was observed in a dose-dependent manner (*p* < 0.01, *p* < 0.001, and *p* < 0.001) in comparison with SC5314 (Figure 6D). As shown in confocal laser scanning microscopy images, the hyphal length became shorter and less numerous as the concentration of FLC increased. Interestingly, the mutant had fewer hyphae at the same concentration of FLC compared with SC5314 (Figure 6B), implying that the *ORF19.5274* gene deletion strengthened the ability of FLC to inhibit biofilm formation in *C. albicans*.

### *Orf19.5274Δ/Δ* intensified oxidative damage of FLC to *Candida albicans* cells

**Figure 7:**
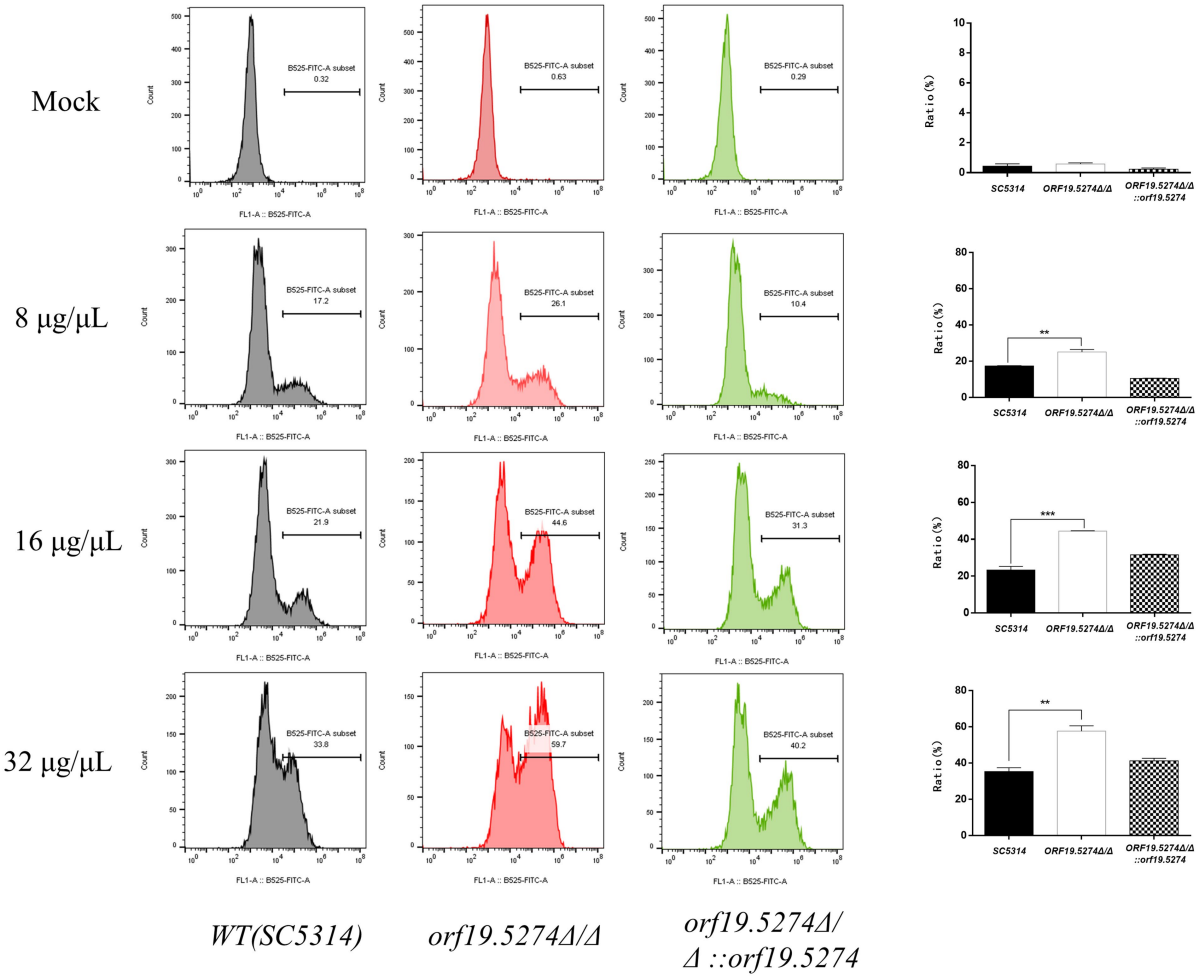
The effect of FLC on the intracellular ROS level of *Candida albicans* cells. The ratio between gated cells and DCFH-DA positive cells in the SC5314, *orf19.5274Δ/Δ*, *orf19.5274Δ/Δ::ORF19.5274* strains, respectively, without FLC treatment or with FLC treatment, was altered.

One of the mechanisms of antifungal action of FLC is the activation of oxidative stress, which causes oxidative. The production of intracellular reactive oxygen species (ROS) is a key factor in oxidative damage, which can induce and regulate apoptosis in yeast or fungal cells (Mahl et al., 2015). In this study, we used DCFH-DA dye to assess the impact of FLC on the intracellular ROS levels of the mutant. The intracellular ROS levels of *C. albicans* without FLC treatment did not differ from those of the parental strain. After 8 h of FLC treatment, the intracellular ROS levels increased with increasing FLC concentration, indicating that FLC could cause the accumulation of intracellular ROS in *C. albicans*. Notably, the level of elevated intracellular ROS in mutant cells was intensified after the addition of the drug compared with that of the parental strain (Figure 7). The deletion of the *ORF19.5274* gene exacerbated the oxidative damage caused by FLC on *C. albicans* cells.

### *Orf19.5274Δ/Δ* showed increased FLC-dependent cell membrane permeability of PI stain

**Figure 8:**
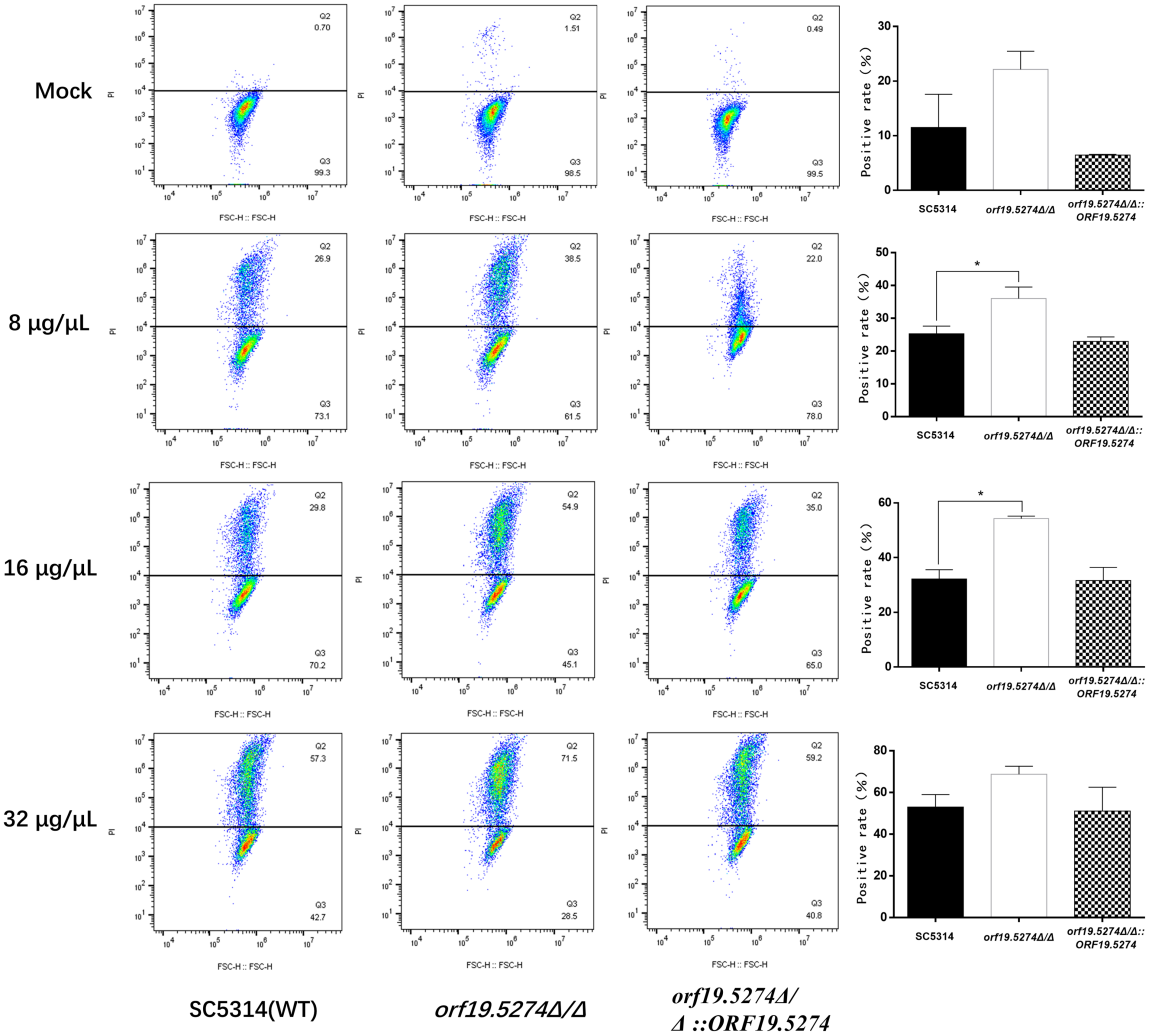
The effect of *orf19.5274Δ/Δ* on the membrane permeability of *Candida albicans* cells. The ratio between gated cells and PI-positive cells in SC5314, *orf19.5274Δ/Δ*, *orf19.5274Δ/Δ::ORF19.5274* strains, respectively, without FLC treatment or with FLC treatment.

FLC is an antifungal drug that induces fungal cell membrane stress, and since the mutant caused increased susceptibility to FLC, we used PI staining to evaluate the effect of this gene deletion on the cell membrane permeability. Without FLC treatment, only a very small percentage of fungal cells were stained with PI, and the percentage of PI-positive cells in the mutant was approximately 1%, which was twice as high as that of the wild and revertant strains, but the difference was not statistically significant (*p* ˃ 0.05). After treatment with different concentrations of FLC, the percentage of PI-positive cells in all three strains gradually increased, and it is noteworthy that the percentage of PI-positive cells in the mutant increased much more compared with the parental and revertant strains (Figure 8). This indicated that the deletion of the *ORF19.5274* gene could cause the enhancement of FLC permeability to the cell membrane of *C. albicans.*

### *Orf19.5274Δ/Δ* improved the efficacy of FLC in the model of *Galleria mellonella* larvae infection

**Figure 9:**
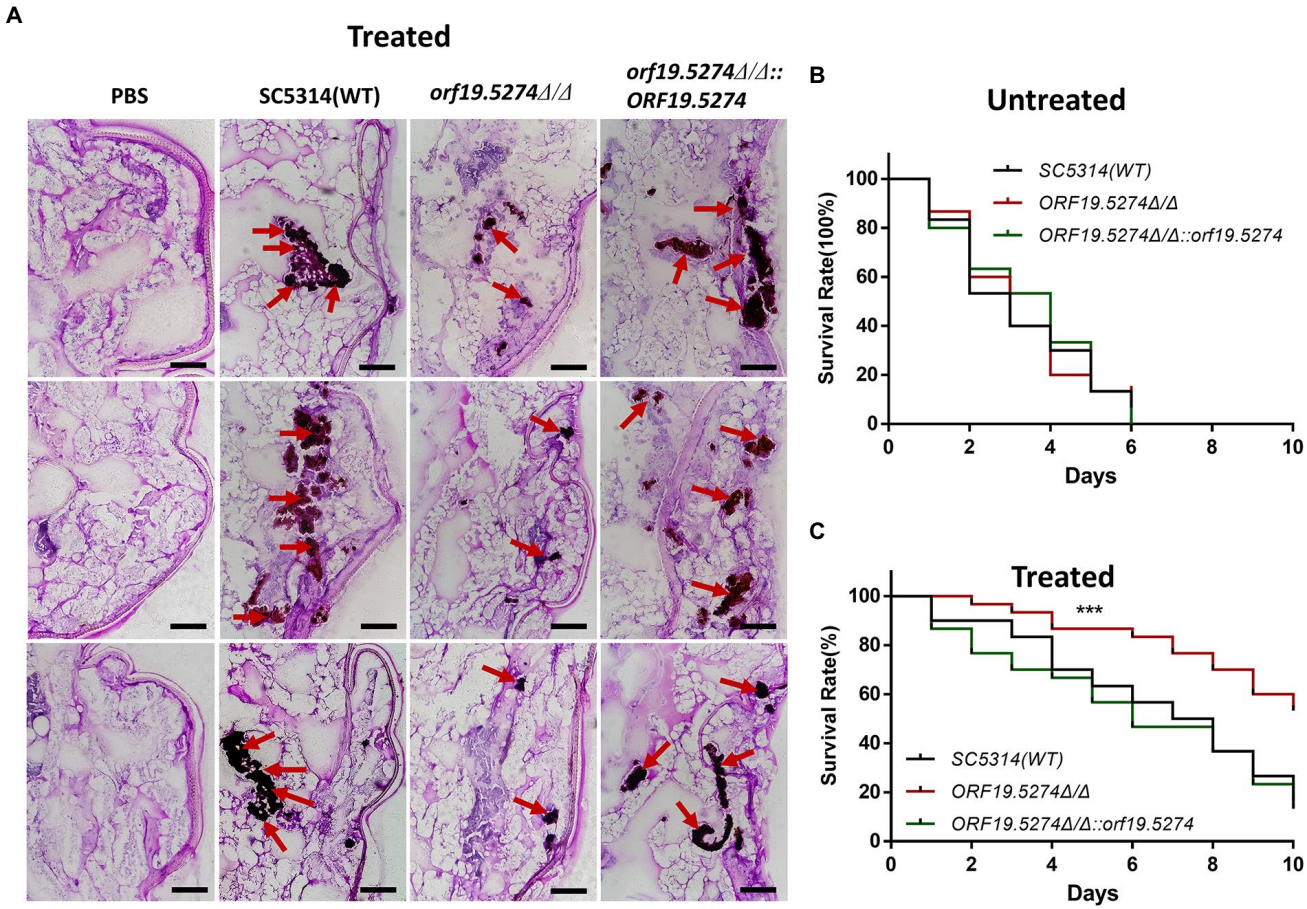
The virulence changes and efficacy of SC5314, *orf19.5274Δ/Δ*, *orf19.5274Δ/Δ::ORF19.5274* in larval infection model of *G. mellonella*. **(A)** PAS section staining of SC5314, *orf19.5274Δ/Δ*, *orf19.5274Δ/Δ::ORF19.5274* strains infected with *G. mellonella* larvae models treated with 64 g/ml FLC. Then, 10 μl of 1 × 10^8^ CFU/ml of fungal cells were injected into the left hind foot of the *G. mellonella* larvae, and the whole worms were taken for frozen sections after 24 h. The red arrows in the pictures show the fungal hypha-yeast mass after staining. **(B)** SC5314, *orf19.5274Δ/Δ*, *orf19.5274Δ/Δ::ORF19.5274* growth curves infected with *G. mellonella* larvae model for 10 days (Log-rank test). **(C)** Survival curves of *G. mellonella* larvae models infected with SC5314, *orf19.5274Δ/Δ*, *orf19.5274Δ/Δ::ORF19.5274* strains for 30 min after treatment with 64 μg/ml FLC, respectively.

*Galleria mellonella* larvae are generally recognized as an animal model for infectious disease studies, and can be used to assess the virulence of pathogenic fungi (Kavanagh and Sheehan, 2018). Since the *ORF19.5274* gene in *C. albicans* was found to inhibit solid hyphal development, we tested whether the virulence of the mutant was reduced using the larval infection model of *G. mellonella*. The survival rate of the mutant was similar to the wild and revertant strains (Figure 9A). Subsequently, we investigated whether the *ORF19.5274* gene deletion improved the efficacy of FLC in the model of *G. mellonella* larval infection. The result showed that after treatment with 64 μg/ml FLC, the mutant significantly accumulated fewer clumps in the insect body than the wild and revertant strains (Figure 9B). The tenth-day survival rate of larvae infected with the mutant strain was 54%, whereas the survival rates of larvae infected with the wild and complementary strains were only 13 and 16%, suggesting that the absence of *ORF19.5274* improves the susceptibility of *C. albicans* to FLC *in vivo*, and therefore the survival rate is greatly increased (Figure 9C). In summary, *orf19.5274Δ/Δ* maintained the variation in susceptibility of *C. albicans* to azoles *in vitro* and *in vivo*.

## Discussion

Fluconazole is a commonly used antifungal drug in the clinical treatment of candida infections, but as an antimicrobial agent, it does not kill pathogenic fungi, providing an opportunity for the development of FLC resistance (Bongomin et al., 2018; Zhang et al., 2019). Given the utility of FLC in the clinical treatment of fungal infections, the prevention of FLC resistance is a topic worthy of investigation. One strategy to improve the efficacy of FLC is to identify synergistic drug targets that enhance the antifungal effect and even reduce the emergence of FLC resistance by making FLC fungicidal (Emami et al., 2019). To investigate whether the new *C. albicans* gene *ORF19.5274* is associated with antifungal drug susceptibility, we constructed the *ORF19.5274* mutant strain and the revertant strain of *C. albicans* using CRISPR-Cas9 gene editing technology to investigate the phenotypic changes and the sensitivity to antifungal drugs, and explored the mechanism of its participation in the sensitivity regulation of FLC.

*Candida albicans* is capable of reversibly switching between yeast, pseudohyphae, and hyphae growth forms, and this morphological plasticity is a key determinant of virulence (Lu et al., 2011). The hyphal form plays a key role in the infection process and promotes tissue penetration and escape from immune cells (Bahn and Sundstrom, 2001; Rocha et al., 2001). Through phenotypic studies, we found that after inducing hyphae in different liquid media for 2 and 5 h respectively, the hyphae formation of the mutant gradually developed from inhibition in the initial stage to hyphal length recovery in the maintenance stage (Figures 5A,C). However, defects in hyphae formation of the mutant in different solid inducing media are significant (Figure 5E). We suggest that in solid inducing media, *C. albicans ORF19.5274* may act as an activator to regulate hyphal-specific genes, while under liquid-induced conditions, its mediated hyphal inhibition does not have a lasting effect. *ORF19.5274*-mediated hyphal inhibition does not stop hyphae from developing and only plays a role at an early stage, which may be due to the influence of multiple forward morphogenesis signaling pathways. The reason for the inconsistent phenotypes of the mutant in solid and liquid inducing media may be that *ORF19.5274* participates in the response to morphogenetic signals in different environments through two distinct mechanisms, involving different ways of cellular response to different environmental stimuli, which deserve further consideration.

By screening the drug-susceptibility phenotype of the mutant strain, it was found that deletion of the *ORF19.5274* increased the sensitivity of *C. albicans* to FLC, which was more obvious in the spot plate experiment. While the sensitivity of the mutant was found to be reduced only 2-fold in the liquid susceptibility test. We suggest this is mainly because the wild-type SC5314 is a FLC-sensitive strain, which has a high sensitivity to FLC, and the liquid susceptibility test is used at a lower concentrations of suspension (~ 10^3^CFU/ml), resulting in an insignificant decrease in its MIC value. The experimental growth curve after FLC treatment confirmed that the sensitivity of the mutant to FLC was increased, and the growth and reproduction rate of the mutant after treatment with FLC was significantly weaker than that of the wild strain (Figure 4B). Therefore, the new gene *ORF19.5274* we studied may also be a potential synergistic target for FLC against *C. albicans*. Based on the functional targets of FLC, we further investigated the reasons for the increased sensitivity of FLC by the deletion of the *ORF19.5274* gene. Ergosterol is a major component of the cell membrane in *C. albicans*, which plays a vital role in maintaining the integrity and fluidity of the membrane and ensuring the proper function of certain membrane-bound enzymes (Pristov and Ghannoum, 2019). Reduced activity of *ERG11*, the target of FLC action, has been reported to lead to increased susceptibility to FLC (Xu et al., 2007), while *C. albicans* strains deficient in squalene epoxidase (*ERG1*), C-8 sterol isomerase (*ERG2*), and C-4 sterol methyl oxidase (*Erg251*) have also increased susceptibility to FLC (Mukhopadhyay et al., 2004; Chen et al., 2018). These enzymes involved in ergosterol biosynthesis may be potential synergistic targets of FLC against *C. albicans*. It has been shown that inhibition of the expression of *ERG1*, *ERG2*, *ERG11*, and *ERG25* genes in *C. albicans* decreases ergosterol levels, thereby increasing membrane fluidity and permeability (Hameed et al., 2011; Danis Vijay et al., 2019). We found that deletion of the *ORF19.5274* gene resulted in the expression of ergosterol synthesis-related genes downregulated (*ERG1*/*ERG2*/*ERG5*/*ERG6*/*ERG11*) by RT-qPCR analysis (Figure 4C). We suspect that the mutant may affect cell membrane permeability by down-regulating genes related to ergosterol synthesis, thereby increasing sensitivity to azoles.

To this end, we have examined changes in the membrane permeability of *C. albicans* cells using PI dyes. Intact cell membranes are not permeable to PI and PI cannot enter the cell to bind to nucleic acids, so the cell cannot be stained. PI itself is not a dye used to show apoptosis specificity; the purpose of PI is to determine damage to the cell membrane and does not correspond to apoptosis. Our data showed that the proportion of PI-positive cells in the mutant strain was approximately two to three times higher than in the wild and complemented strains, but the difference was not statistically significant. Whereas the proportion of PI-positive cells in the mutant strain after FLC treatment was significantly more than in the wild and revertant strains (Figure 8), indicating that the mutant cells showed FLC-dependent increased cell membrane permeability. Combined with the fact that the mutant strain down-regulated the transcript levels of genes related to ergosterol synthesis, it is suggested that the increased sensitivity of the mutant to FLC is related to the increased cell membrane permeability caused by reduced ergosterol levels. Besides, studies have shown that the antifungal activity of FLC is related to oxidative damage induced by the accumulation of endogenous ROS, which is essential for normal cell function and leads to apoptosis through different pathways (Atriwal et al., 2021). Here, the mutant did not affect the intracellular ROS levels, suggesting that the increased sensitivity to FLC or defects in solid hyphal development due to deletion of *ORF19.5274* was not due to impaired oxidative stress, as confirmed by spot plate assays with H_2_O_2_ (Figure 3). However, the mutant significantly increased the oxidative stress response of FLC against *C. albicans*. The elevated level of intracellular ROS after FLC treatment was higher than that of wild and revertant strains, which exacerbated the oxidative damage to the cells (Figure 7). Above all, these results imply that *ORF19.5274* deletion could enhance the anti-*C. albicans* effect of FLC *in vitro* by increasing FLC-dependent cell membrane permeability through impeding ergosterol synthesis or exacerbating oxidative damage through elevating endogenous ROS levels.

Hyphae are an important part of biofilms and play a key role in building a robust three-dimensional biofilm structure. There is growing evidence that the development of the *C. albicans* biofilm is a highly regulated and coordinated process that can be divided into four main stages: adhesion, proliferation, maturation, and diffusion (Gulati and Nobile, 2016; Lohse et al., 2018). The formation of mature biofilms in *C. albicans* is closely related to its drug resistance. Three main factors have been reported to be involved in the resistance properties of *C. albicans* biofilms (Gulati and Nobile, 2016). One of the drug-resistant properties of *C. albicans* biofilms is the presence of persistent cells. These dispersed cells from biofilms have distinct characteristics compared with planktonic cells. For example, the dispersed cells have increased adherence properties and have a higher capacity to form biofilms relative to planktonic cells. From a clinical perspective, increased tolerance of cells within the biofilm to antifungal drugs and protection from host immune defenses are the two primary consequences of biofilms that negatively affect the treatment of infected patients (Kernien et al., 2018). The biofilm extracellular matrix is a second major contributor to antifungal drug resistance in *C. albicans* biofilms. The matrix acts as both a physical barrier to drug penetration and as a stabilizer of the overall architecture of the biofilms (Nett et al., 2007; Nobile and Johnson, 2015), isolating antifungal molecules and preventing them from penetrating deeper into the biofilm (Pierce et al., 2013; Possamai Rossatto et al., 2021). A third important resistance property is the biofilm-regulated efflux pumps. The immediate upregulation of efflux pumps that occurs in the early stage of biofilm development is a key contributor to the early recalcitrance of biofilms to antifungal agents (Nobile et al., 2012). In addition, changes in the composition of sterols in biofilms also seem to be associated with drug resistance. Biofilm cells have a distinctly lower concentration of ergosterol in their cell membranes than do planktonic cells (Mukherjee et al., 2003). The finding suggests that mature biofilms are less dependent on ergosterol for membrane fluidity, potentially limiting the effectiveness of ergosterol-targeted drugs (FLC). As mentioned above, biofilm resistance to standard antifungal drugs, especially FLC, which is the most widely used clinically, is multifactorial, and there is currently no biofilm-specific drug available to treat any biofilm-based microbial infection. Therefore, in our XTT experimental results, we can see that FLC has little effect on the removal of mature biofilms. At the same time, the mutant strains have not been effective in improving FLC ‘s ability to clear mature biofilms (Figures 6A,C), possibly because the low effect of FLC clearing mature biofilms counteracts the effect of the mutant’s increased sensitivity to FLC. These biofilm-specific properties make it particularly challenging to develop effective treatments for biofilm infections. We also examined the ability of FLC to inhibit the formation of *C. albicans* biofilms and found that FLC inhibits approximately 50% of biofilm metabolic activity at concentrations of 128 μg/ml (Figures 6B,D), which is more effective than treating mature biofilms. We suggest that this is related to the properties of the mature biofilm itself, as described earlier. The initial yeast cell co-culture with FLC keeps fungal cells under drug stress and inhibits the natural development process of the biofilm, which is incomplete in structure and does not fully possess the drug resistance characteristics of mature biofilm, so FLC is relatively effective. Furthermore, the mutant increased the ability of FLC to inhibit biofilm formation in a dose-dependent manner. This may be due to the fact that FLC is relatively effective against immature biofilms. The effect of increasing sensitivity of mutant to FLC is maintained, so that the effectiveness of the mutant in inhibiting biofilm formation of FLC is also increased. Although the mutant did not alter FLC’s ability to clear mature biofilm, the enhancement of FLC’s ability to inhibit biofilm formation gave us confidence in finding synergistic anti-biofilm targets for FLC.

*Galleria mellonella* larvae infection model is a relatively mature model for simulating human pathogenic infection. Its innate immune system is similar to the mammals, making it an effective model for the study of pathogen infection (Cutuli et al., 2019). Compared with the most widely used mouse models, they are inexpensive, readily available, easier to construct, require little in the way of laboratory conditions, and have no ethical concerns (Rossoni et al., 2019). Therefore, increasing studies have considered the larvae as the second choice for the infective model *in vivo*. In our study, deletion of *ORF19.5274* did not affect the virulence of *C. albicans* (Figure 9B), but significantly improved the efficacy of FLC in the *G. mellonella* larval infection model (Figure 9C). In addition, PAS staining showed that the fungal colony size of the mutant strain was significantly less than the wild-type and complementary strains, which was consistent with the survival curve of the treatment group. The results indicated that deletion of the *ORF19.5274* gene could enhance the anti-*Candida albicans* effect of FLC *in vitro* and *in vivo*.

## Conclusion

It is very difficult to find an effective, low-toxic, and non-drug-resistant fungicide that can be applied in clinical practice. Therefore, to look for more usage of the azoles widely used in clinics is a breakthrough point, which requires finding more azole targets to kill the fungus more effectively. Deletion of the new gene *ORF19.5274* was described herein, although it does not make FLC fungicidal, it could increase FLC’s ability to inhibit the survival of *C. albicans*. Overall, the *ORF19.5274* gene may be a potential target for reducing the resistance of *C. albicans* to azoles. Despite the identification of this new gene, there are still many issues raised by this paper. Our study found that mutant cells react differently in liquid and solid hyphal induing medium. The transcriptional expression of the relevant hyphal-specific genes described in this paper are closely related to hyphal development. Besides, the deeper cellular response mechanisms and regulation need to be further studied. Bioinformatics analysis and prediction only give us a direction for future research, we will continue to investigate whether *ORF19.5274* indeed function as a transcription factor, verify the changes of its upstream and downstream genes and their general regulatory processes through transcriptomics and experiments, and determine whether this gene is involved in the regulation of a specific pathway in *Candida albicans*.

## Data availability statement

The original contributions presented in the study are included in the article/Supplementary material, further inquiries can be directed to the corresponding author.

## Author contributions

MH initiated the study and drafted the manuscript. MH and GG were in charge of the conception and design of the study and wrote the first draft of the manuscript. MH, LY, LZ, CS, and CT performed the experiments and interpreted the data. JP, WZ, and ZJ contributed to the study data analysis and translation also providing technical support. All authors contributed to the article and approved the submitted version.

## Funding

This work was supported by the National Natural Science Foundation of China (no. 82060381) and Science and Technology Planning Project of Guizhou Province [ZK (2022) general project 345].

## Conflict of interest

The authors declare that the research was conducted in the absence of any commercial or financial relationships that could be construed as a potential conflict of interest.

## Publisher’s note

All claims expressed in this article are solely those of the authors and do not necessarily represent those of their affiliated organizations, or those of the publisher, the editors and the reviewers. Any product that may be evaluated in this article, or claim that may be made by its manufacturer, is not guaranteed or endorsed by the publisher.

## Footnotes

^1^ www.benchling.com
