# Supplementary material for: Identification and functional characterization of ORF19.5274, a novel gene involved in both azoles susceptibility and hypha development in Candida albicans
Source: Front Microbiol. 2022 Oct 3;13:990318. doi: 10.3389/fmicb.2022.990318 (PMC9575988; doi:10.3389/fmicb.2022.990318)
Supplement: Supplementary file 1 [file Table_1.DOCX]

Supplementary Material

# Tab S1. Primes used for *orf19.5274Δ/Δ* deletion and the detection of transcriptional levels of ERG genes and other genes related to hyphal and biofilm regulation

| Primer | | Sequence |
| --- | --- | --- |
| gRNA | cgtaaactatttttaatttgGGTTGACCAGAAGCACCCAGgttttagagctagaaatag | |
| *Orf19.5274*-2 | gtccaaccaacaccattaagat | |
| *Orf19.5274*-3 | CCTCATGTCGAGCACTCGTCTCGgatagcaatttagtaggtat | |
| *Orf19.5274*-4 | CGAGACGAGTGCTCGACATGAGGgattggatagttattgatt | |
| *Orf19.5274*-5 | ggcgataacaaagggaaaattc | |
| *Orf19.5274*-6 | ctgcaacaaccacaaagacct | |
| *Orf19.5274*-7 | cctagcttggccattaggatt | |
| ERG1-F | gaccgaatagaaggcaacac | |
| ERG1-R | ggacgctgttatcaacacat | |
| ERG2-F | gccttgagccaattctaagg | |
| ERG2-R | ctggtgaacaaagagcagct | |
| ERG5-F | cccaacataccagtgaacaa | |
| ERG5-R | cctgaagtttatgatgagcc | |
| ERG6-F | ccatcaccgacttcaatacc | |
| ERG6-R | gctgtttatgccattgaagc | |
| ERG11-F | cacgtctccaataatgaggt | |
| ERG11-R | catggggttgccaatgttat | |
| EFG1-F | TATGCCCCAGCAAACAACTG | |
| EFG1-R | TTGTTGTCCTGCTGTCTGTC | |
| CPH1-F | AAACCCAGAAGCGTCAT | |
| CPH1-R | ACCCAGCAGAAATACCG | |
| HWP1-F | TGGTGCTATTACTATTCCGG | |
| HWP1-R | CAATAATAGCAGCACCGAA | |
| ALS3-F | tcacctgcctgaaattgaca | |
| ALS3-R  CDR1-F  CDR1-R  CDR2-F  CDR2-R  MDR1-F  MDR1-R | Tggaatgctgttttgggttg  ctaagatgtcgtcgcaag  ctgctgacgagtcatctt  cacgtctttgtcgcaaca  tatgtcggacatgtggct  ccacatcagcaacacttg  tatggtgttggcccattg | |


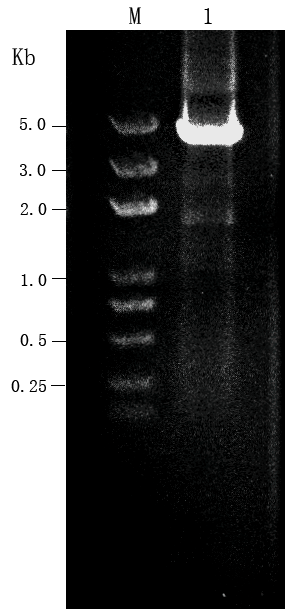


**FigS1. Linearized pADH100 plasmid**

**
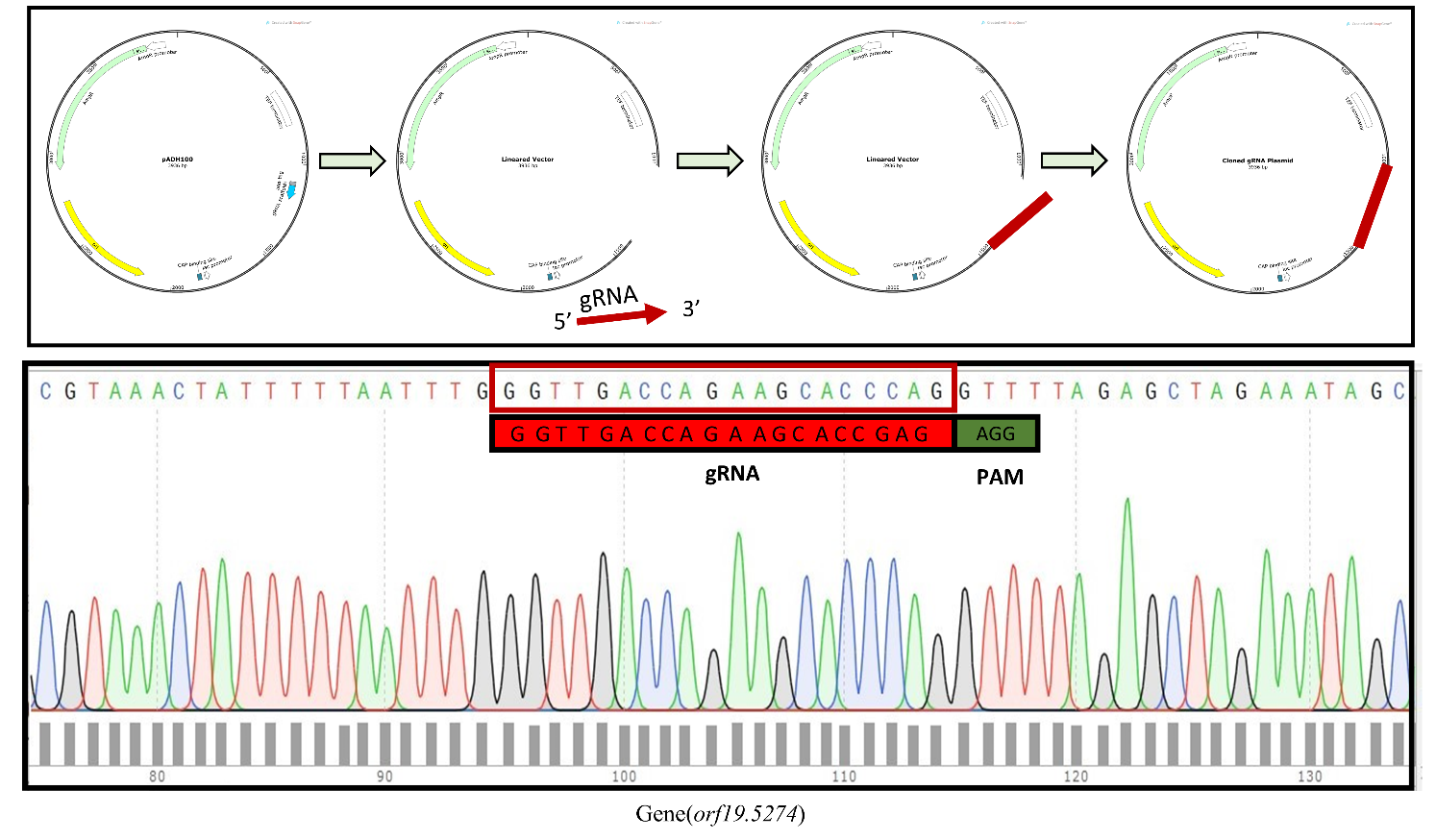
**

**FigS2.** Construction and sequencing of pADH100-gRNA

**FigS3.** Mean fluorescence intensity of CLSM images of mature biofilm was analyzed by Image-Pro Plus 6.0 software. Scale bars represent the standard deviation (SD) (n=3). ** *p* < 0.01. The biofilms were stained with viability indicators (SYTO9 and PI). Live cells represent SYTO9 and dead cells represent PI.

**FigS3.** Expression of genes related to transport in SC5314, mutant, and revertant strains, respectively. The expression of the above genes in SC5314 was normalized and the remaining strains were controlled by the wild-type, and the 2-ΔΔCt values were calculated. Three replicates were performed for each gene.
